# Shared genetic architecture between COVID-19 and irritable bowel syndrome: a large-scale genome-wide cross-trait analysis

**DOI:** 10.3389/fimmu.2024.1442693

**Published:** 2024-11-15

**Authors:** Xianqiang Liu, Dingchang Li, Wenxing Gao, Hao Liu, Peng Chen, Yingjie Zhao, Wen Zhao, Guanglong Dong

**Affiliations:** ^1^ Medical School of Chinese PLA, Beijing, China; ^2^ Department of General Surgery, The First Medical Center, Chinese PLA General Hospital, Beijing, China; ^3^ School of Medicine, Nankai University, Tianjin, China

**Keywords:** COVID-19, irritable bowel syndrome, genome-wide association study, MTAG, LDSC

## Abstract

**Background:**

It has been reported that COVID-19 patients have an increased risk of developing IBS; however, the underlying genetic mechanisms of these associations remain largely unknown. The aim of this study was to investigate potential shared SNPs, genes, proteins, and biological pathways between COVID-19 and IBS by assessing pairwise genetic correlations and cross-trait genetic analysis.

**Materials and methods:**

We assessed the genetic correlation between three COVID-19 phenotypes and IBS using linkage disequilibrium score regression (LDSC) and high-definition likelihood (HDL) methods. Two different sources of IBS data were combined using METAL, and the Multi-trait analysis of GWAS (MTAG) method was applied for multi-trait analysis to enhance statistical robustness and discover new genetic associations. Independent risk loci were examined using genome-wide complex trait analysis (GCTA)-conditional and joint analysis (COJO), multi-marker analysis of genomic annotation (MAGMA), and functional mapping and annotation (FUMA), integrating various QTL information and methods to further identify risk genes and proteins. Gene set variation analysis (GSVA) was employed to compute pleiotropic gene scores, and combined with immune infiltration algorithms, IBS patients were categorized into high and low immune infiltration groups.

**Results:**

We found a positive genetic correlation between COVID-19 infection, COVID-19 hospitalization, and IBS. Subsequent multi-trait analysis identified nine significantly associated genomic loci. Among these, eight genetic variants were closely related to the comorbidity of IBS and COVID-19. The study also highlighted four genes and 231 proteins associated with the susceptibility to IBS identified through various analytical strategies and a stratification approach for IBS risk populations.

**Conclusions:**

Our study reveals a shared genetic architecture between these two diseases, providing new insights into potential biological mechanisms and laying the groundwork for more effective interventions.

## 1 Introduction

Since January 2020, the coronavirus disease 2019 (COVID-19) pandemic, driven by the highly contagious severe acute respiratory syndrome coronavirus 2 (SARS-CoV-2), has affected nearly 600 million individuals worldwide, exerting substantial strain on global healthcare systems (1–3). SARS-CoV-2, an RNA virus, gains entry into host cells through the angiotensin-converting enzyme 2 (ACE2) receptor and its spike protein (4). Although ACE2 receptors are predominantly expressed in the lung parenchyma, they are also distributed in various other cell types, including those in the oral and nasal mucosa, gastrointestinal tract, pancreas, liver, and vascular endothelium (5). Upon infiltrating these cells, SARS-CoV-2 triggers an inflammatory response, leading to the activation of immune cells (4). Persistent or emerging symptoms following SARS-CoV-2 infection, such as lung damage, mental health issues, gastrointestinal symptoms, and systemic conditions, are collectively known as “long COVID-19” (6, 7). Given the expression of ACE2 receptors in the gastrointestinal epithelium, COVID-19 can substantially impact gastrointestinal function (5, 8, 9). Recent research (10) indicates a higher prevalence of irritable bowel syndrome (IBS) within a year post-infection compared to uninfected controls defined as COVID-negative according to the WHO criteria. IBS, a chronic gastrointestinal disorder, is characterized by symptoms such as bloating, abdominal pain, and changes in bowel habits (11). This condition notably compromises the quality of life and social capabilities of those affected, with its global prevalence estimated to be between 9% and 23% (12, 13). A thorough investigation of the relationship between COVID-19 and IBS could aid in developing effective policies and personalized treatments, thus controlling the spread of the pandemic and reducing the societal healthcare burden.

Research limited to a single disease may fail to identify critical genetic loci and molecular regulatory mechanisms. Thus, adopting multi-trait analysis methods is necessary to expand the phenotype spectrum under investigation, identify associated risk loci, and delve into the shared genetic etiologies among different diseases (14). Shared genetic etiologies indicate potential pleiotropy, which often represents genetic confounding factors linking traits (15, 16). Hence, it is suggested to implement cross-trait analysis, which utilizes the correlation between the genome-wide association studies (GWAS) data of COVID-19 and IBS to explore pleiotropic genetic variations or loci across multiple traits (17, 18). These pleiotropic loci could serve as potential intervention targets, offering opportunities for simultaneous prevention or treatment of these diseases.

**Figure 1:**
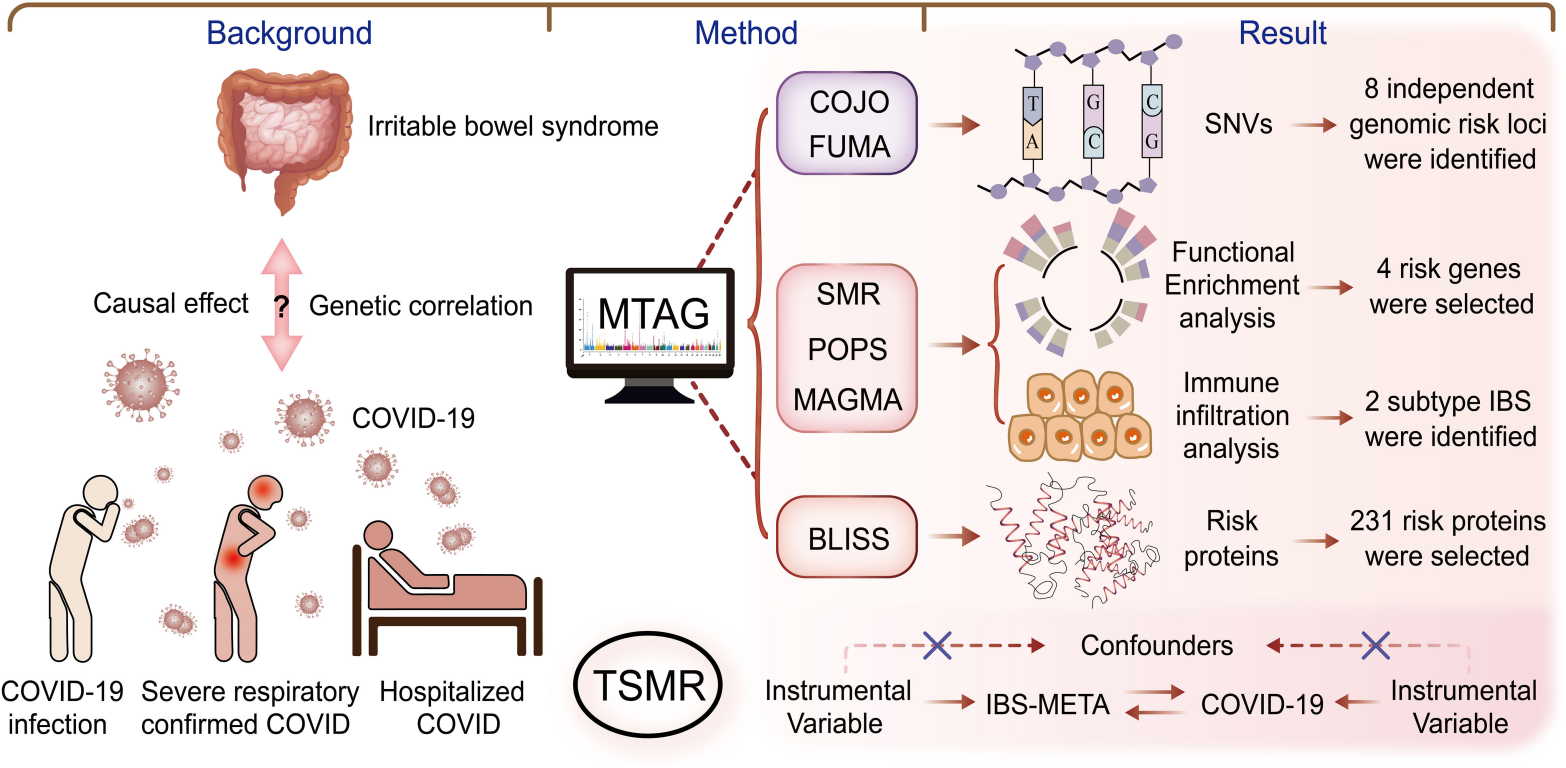
Overall study design. This study investigated the genetic correlation between IBS and COVID-19, using methods such as COJO, POPS, MAGMA, SMR, and BLISS to explore connections from SNVs to genes, proteins, and signaling pathways. Our goal was to identify potential therapeutic targets for treating IBS in long COVID patients. BLISS, Biomarker Imputation from Summary Statistics; COJO, Conditional and joint analysis; HDL, High-definition likelihood; LDSC, Linkage disequilibrium score regression; MAGMA, Multi-marker analysis of genomic annotation; MTAG, Multi-trait analysis of GWAS; POPS, Polygenic priority score; SMR, Summary-based Mendelian Randomization.

In this genome-wide association study, large-scale GWAS summary data were utilized to analyze IBS and COVID-19 datasets from various sources using an array of statistical genetic methods. The pleiotropic associations were examined sequentially at the single nucleotide variant (SNV), gene, and protein levels, along with biological pathways, to uncover potential shared genetic etiologies. Initially, linkage disequilibrium score regression (LDSC) and high-definition likelihood (HDL) were employed to evaluate genetic correlations. Within the pleiotropic analysis framework, multi-trait analysis of GWAS (MTAG) and genome-wide complex trait analysis (GCTA)-conditional and joint analysis (COJO) were applied to identify shared pleiotropic genetic loci at the SNV level for both IBS and COVID-19. Subsequently, multi-marker analysis of genomic annotation (MAGMA), polygenic priority scoring (PoPS), and summary data-based Mendelian randomization (SMR) analyses were conducted at the gene level to identify candidate pleiotropic genes. At the protein level, BLISS was utilized to determine risk proteins. Furthermore, gene ontology (GO) biological processes and Kyoto Encyclopedia of Genes and Genomes (KEGG) pathways were explored for enrichment. Finally, IBS transcriptome data were integrated, and Gene Set Variation Analysis (GSVA) was used to investigate the expression of pleiotropic genes in IBS subtypes, identifying the immune characteristics of high- and low-risk IBS groups. **Figure 1** illustrates the overall study design.

## 2 Data sources and methods

A series of comprehensive GWAS summary data were utilized in this study. Given the limited availability of GWAS data from non-European ancestries, GWAS summary data were predominantly sourced from publicly available datasets of European ancestry. IBS GWAS data were derived from GCST90016564, encompassing 53,400 patients and 433,201 controls. An additional IBS GWAS dataset was obtained from Finn-IBS, based on a Finnish database study that included 339,710 individuals (10,329 cases and 329,381 controls).

The most recent COVID-19 phenotype summary statistics, which include susceptibility, hospitalization, and severe clinical outcomes, were collected from the COVID-19 host genetics initiative (HGI) GWAS meta-analysis (Round 7) (19). The confirmation of COVID-19 cases was based on laboratory-confirmed tests, electronic health records, diagnoses made by physicians, or patient self-reports of SARS-CoV-2 infection. Susceptibility results compared COVID-19 cases (N = 122,616) with control individuals with no history of COVID-19 (N = 2,475,240). Individuals classified as hospitalized COVID-19 cases were those with a laboratory-confirmed SARS-CoV-2 infection or those admitted to the hospital due to COVID-19-related symptoms. The study compared the hospitalization outcomes of these patients (N = 32,519) with controls who were not hospitalized for COVID-19 (N = 2,062,805). Individuals classified as severe COVID-19 cases were those hospitalized who needed respiratory assistance, including intubation, continuous positive airway pressure (CPAP), bilevel positive airway pressure (BiPAP), continuous external negative pressure, or high-flow nasal cannula. The severity evaluations involved a comparison between these severe COVID-19 cases (N = 13,769) and individuals who did not require such severe medical interventions (including those without COVID-19) (N = 1,072,442). The dataset from the COVID-19 GWAS was adjusted for variables such as age, sex, age × sex, principal components, and covariates specific to the study, as outlined by the GWAS researchers (20). All GWAS data sources were listed in **Supplementary Table S1**.

To elucidate the genetic architecture of IBS, quantitative trait loci (QTL) data were integrated, including expression QTLs (eQTLs) from 54 specific tissues such as gastrointestinal tissue and blood, as well as plasma protein QTLs (pQTLs) from three sources (decode, UKBPP, ARIC). The eQTL analysis included blood eQTL information from the extensive eQTLGen consortium database, which documented trait-associated SNPs in a cohort of 31,684 individuals (21). Furthermore, plasma pQTL information was sourced from deCODE, which performed extensive protein measurements in 35,559 Icelandic participants, focusing on 4,907 plasma proteins (22). The plasma pQTL data also included information on 4,953 plasma proteins from the ARIC database (http://nilanjanchatterjeelab.org/pwas/) and 2,923 plasma proteins from the UKBPP project, which involved 54,219 participants from the UK Biobank (23).

### 2.1 Statistical analysis

During the analysis phase of this study, we meticulously excluded SNVs within the major histocompatibility complex (MHC) region on chromosome 6 (25–35 Mb) to mitigate potential confounding effects. In addition, we removed SNPs with a minor allele frequency of <0.01 and those with duplicated or missing reference cluster IDs from each GWAS summary dataset for subsequent analysis. Data aligned to the GRCh38 reference were converted to GRCh37 using the liftOver tool for consistency (24).

#### 2.1.1 Assessment of genetic correlations between IBS and COVID-19

We conducted LDSC on two sources of IBS and COVID-19 patients, hospitalized patients, and severe patients from GWAS data (25). In LDSC, constraints were not imposed on the intercept, as sample overlap affects only the intercept, not the regression slope or genetic correlation. This approach allowed for the acknowledgment of residual confounding factors and revealed potential sample overlap in the two GWAS datasets. We also employed the HDL method to assess genetic correlations. The HDL method extends the LDSC approach by modeling the relationship between Z-statistics covariance for trait pairs across multiple SNVs and the full matrix of cross-SNV LD scores. To ensure the accuracy and reliability of the analysis, all data were subjected to Benjamini and Hochberg (BH) adjustment for multiple comparisons, with an adjusted *P*-value of <0.05 indicating statistical significance.

#### 2.1.2 Meta-analysis of GWASs

Meta-analysis was performed to combine data from the two IBS datasets. Given the potential sample overlap between the datasets, Metasoft was used to evaluate heterogeneity (I^2^) and *P*-values based on Cochran’s Q test (*P*_het). When heterogeneity was present (I^2^ ≥ 50 or *P*_het < 0.05), *P*-values from the random-effect model calculated using RE2C were considered (26, 27).

#### 2.1.3 Multi-trait analysis of GWAS summary statistics

Building on the results from the previous stage of research, we continued with a multi-trait analysis, conducting a cross-analysis of META-IBS with COVID-19 phenotypes that exhibited significant genetic correlation. MTAG combines the summary statistics from GWAS of genetically correlated traits into a meta-analysis, accounting for genetic correlation and sample overlap, to maximize the power to identify loci associated with the target traits (28). We combined the GWAS data of phenotypes that exhibited a genetic correlation with IBS to produce MTAG-IBS. The genome-wide significance threshold was set at a *P*-value of < 5 × 10^-8^ to ensure accurate identification of correlations.

#### 2.1.4 Identification of genetic risk factors for IBS

##### 2.1.4.1 Identification of independent risk loci

To identify genomic risk factors for IBS, we detected distinct, independent signals within the genomic loci associated with MTAG-IBS using the stepwise model selection framework provided by GCTA-COJO (29, 30). The analysis was limited to multiallelic variants that exhibited significant correlations (*P*.mtag < 5 × 10^-8^) within previously established genomic risk loci, and additional signals were confirmed based on a joint *P*-value threshold of < 5 × 10^-8^. This analysis benefitted from the reference dataset provided by the third iteration of the 1000 Genomes Project, particularly the European ancestry cohort (25). Based on the results of MTAG, the identified pleiotropic loci were mapped to neighboring genes to investigate their shared biological mechanisms. The functional mapping and annotation (FUMA) platform (31) was used to delineate the genomic risk loci through functional annotation of the variants. The maximum *P*-value for lead SNVs was set at <5 × 10^-8^, whereas the broader significance threshold was set at *P*-values of <0.05. Independent and lead SNVs were identified based on an r2 threshold of <0.6 and <0.1 within a 1-Mb radius, respectively. Genomic risk loci were defined by merging areas wherein lead SNVs were <250 kb apart. SNVs validated through GCTA-COJO and FUMA analyses were identified as risk factors for IBS.

##### 2.1.4.2 Genetic insights into IBS

In the integrated analysis aimed at revealing the genetic basis of IBS, MAGMA and POPS (32, 33) were used to identify and prioritize relevant genes, with *P*-values adjusted using the BH procedure in each method. Genes with false discovery rate (FDR)-adjusted *P*-values of <0.05 and those consistently identified using both methods were considered significant risk factors. MAGMA enables gene-centered analysis based on extensive data from protein-coding genes and can be integrated with POPS to prioritize enriched genes. In particular, this approach integrates GWAS summary data with expression profiles and biological pathways, with a POPS score of >1 indicating candidate genetic risk factors.

To examine the genetic composition of individual IBS cases, SMR was performed using the GWAS summary data of patients with IBS and the eQTL data of various tissues and cell types (34). LD scores from the European ancestry cohort of the 1000 Genomes Project (25) were used to investigate the relationship between gene expression and IBS. The inclusion criteria were as follows: FDR-adjusted *P*-value < 0.05; heterogeneity (HEIDI) > 0.01.

To explore the biological relevance of genes associated with COVID-19 combined with IBS, we performed genomic enrichment analysis. This analysis utilized data from the Molecular Signatures Database (MSigDB) (35), and significant biological pathways were identified using the ClusterProfiler tool, following adjustment for multiple testing (36).

##### 2.1.4.3 Proteomic insights into IBS

The “Biomarker Level Inference from Summary Statistics” (BLISS) method was used to examine the complex proteomic landscapes of IBS and COVID-19. Traditional proteome-wide association study (PWAS) models depend on detailed individual-level proteomic data. This dependence often limits the ability to utilize the vast amount of summary-level pQTL data available publicly (37). In contrast to traditional PWAS models, the BLISS method represents a novel strategy for constructing protein imputation models directly from summary-level pQTL data. In this study, the BLISS method was used to generate extensive European PWAS models using pQTL data from large-sample UKB, deCODE, and ARIC studies (37). Proteins with an FDR-adjusted P-value of <0.05 were identified as significant risk factors, indicating their potential key role in the pathophysiology of IBS and COVID-19.

##### 2.1.4.4 Two-sample MR

MR, a type of instrumental variable analysis widely used for causal inference, was used to examine causal relationships between IBS and COVID-19. Exposure-related SNPs were used as instruments (38, 39), and GWAS summary data were used to identify variants associated with IBS and COVID-19 (*P*-value < 5.0 × 10^-8^). The IVW method was primarily used, with LD and physical distance thresholds being set at 0.001 and 10 Mb, respectively. To ensure the robustness of the instrumental variables, the determination coefficient r^2^ and F-statistic were calculated, and SNPs with an F-value of >10 were selected. Additionally, the MR-PRESSO method (repeated 1000 times) was used to detect outliers (15), which were removed for re-evaluation. To ensure the reliability and robustness of the results, we conducted sensitivity analyses, including Cochran’s Q test, MR-Egger intercept test, funnel plot, and leave-one-out analysis. Cochran’s Q test was used to assess potential heterogeneity to determine if the variability of the independent variable could lead to different outcomes. If a p-value of ≤0.05 indicated the presence of heterogeneity, a random-effects IVW MR analysis was used (40). The purpose of the MR-Egger intercept test was to detect the potential presence of directional pleiotropy, a phenomenon where the independent variable influences the outcome through pathways other than exposure (41). A funnel plot was used for visual inspection of the symmetry of the distribution of effect estimates. Any obvious asymmetry in the funnel plot may indicate heterogeneity. Leave-one-out analysis involved systematically excluding each SNP and then re-evaluating the effect estimates to determine the reliability and robustness of the results by assessing the impact of each SNP on the overall outcome.

##### 2.1.4.5 GSVA and Immune cell infiltration analysis

To estimate the infiltration levels of various immune cells in IBS patients, we utilised mRNA expression data from four datasets containing IBS patients—GSE13367, GSE14841, GSE36701, and E-TABM-176—obtained from the Gene Expression Omnibus (GEO) and EMBL databases (42). These datasets included a total of 188 IBS patients. The datasets were merged into a single normalised expression matrix using the “combat” function in the “sva” package (version 3.42.0), effectively eliminating batch effects, which was confirmed via principal component analysis (43). Risk-related genes were quantified using the single-sample gene set enrichment analysis (ssGSEA) algorithm in the “GSVA” (version 1.24.0) R package. Based on the results of ssGSEA, patients in the IBS cohort were divided into high- and low-immune-infiltration groups. Subsequently, the “deconvolute_xcell” function in the “immunedeconv” R package (version 2.1.0) (44) was used to evaluate the proportion of 64 types of immune and stromal cells in the two groups.

## 3 Results

### 3.1 Genetic relationship between IBS and COVID-19

GWAS summary data from two IBS datasets were subjected to LDSC and HDL analysis to examine the genetic relationship between IBS and COVID-19 patients, hospitalized patients, and severe patients. After stringent BH correction, significant genetic correlations were observed between IBS and two COVID-19 phenotypes (COVID-19 infection and Hospitalized covid) in both IBS datasets (**Table 1**). Subsequently, GWAS summary data from the two datasets were integrated to obtain the consolidated META-IBS dataset. This dataset included 9,781,012 validated SNPs. After SNPs within the MHC region were excluded, a total of 8,987 statistically significant genetic loci were identified.

**Table 1:** Genetic correlation analysis results.

Trait pair	LDSC		HDL	
	rg (SE)	*P*- FDR	rg (SE)	*P*- FDR
COVID-19 infection-IBS (dataset1)	0.210 (0.082)	0.016	0.231 (0.091)	0.018
COVID-19 infection-IBS (dataset2)	0.135 (0.050)	0.016	0.183 (0.052)	0.003
Hospitalized COVID-19 -IBS (dataset1)	0.177 (0.069)	0.016	0.160 (0.073)	0.035
Hospitalized COVID-19 - IBS (dataset2)	0.117 (0.043)	0.016	0.170 (0.051)	0.003
Very severe respiratory confirmed COVID-19 -IBS (dataset1)	0.087 (0.079)	0.263	0.134 (0.087)	0.127
Very severe respiratory confirmed COVID-19- IBS (dataset2)	0.085 (0.044)	0.060	0.150 (0.048)	0.004

The genetic correlation was estimated using the LDSC method, while the genetic overlap was assessed using the HDL method. A Benjamini and Hochberg (BH)–corrected significance threshold was set to account for multiple comparisons. Notably, we observed a significant genetic correlation between irritable bowel syndrome (IBS) and COVID-19-infected, hospitalized patients. These findings highlight a potential common genetic factor between IBS and COVID-19. LDSC, linkage disequilibrium score regression; HDL, high-definition likelihood; rg, regression; dataset1, FinnGen-IBS; dataset2: GCST90016564-IBS.

Subsequently, GWAS summary data from the two datasets were integrated to obtain the consolidated META-IBS dataset. This dataset included 10,634,628 validated SNPs. After SNPs within the MHC region were excluded, a total of 1,166 statistically significant genetic loci were identified.

### 3.2 Bidirectional Mendelian randomization study

A bidirectional MR study was conducted to delineate the potential causal relationship between COVID-19 and IBS, and the findings revealed no causal association between the two conditions (**Supplementary Table S2**). The random effects inverse variance weighted (IVW) results demonstrated no genetic causal relationship for either COVID-19 susceptibility (*p =* 0.616, OR = 1.021, 95% CI = 0.940-1.109) or COVID-19 hospitalization (*p =* 0.609, OR = 1.007, 95% CI = 0.979-1.036). In the MR analysis relating to IBS, SARS-CoV-2 infection, and hospitalization events, no heterogeneity was detected, as evidenced by Cochran’s Q statistic (MR-IVW) and Rucker’s Q statistic (MR-Egger) (*p >* 0.05). Similarly, the MR Egger intercept test did not detect horizontal pleiotropy in the analysis of these conditions (*p >* 0.05). Moreover, the MR-PRESSO global test further corroborated the non-existence of horizontal pleiotropy (*p >* 0.05).

### 3.3 Genetic landscape of IBS identified through multi-trait analysis

The genetic landscape of IBS was examined using the META-IBS dataset and the GWAS summary data of two COVID phenotypes. The MTAG method was used to generate an enhanced dataset (MTAG-IBS), which included 6,941,121 SNPs. A total of 241 SNPs were identified in the MTAG-IBS dataset (*P*.mtag < 5× 10^-8^).

### 3.4 Genetic markers associated with COVID-19 and IBS comorbidity

The advanced GCTA-COJO tool was used for stepwise model selection in the MTAG-IBS dataset. A total of 9 SNVs were identified through this rigorous process (**Supplementary Table S3**). Subsequently, Based on MTAG results, the FUMA platform revealed 8 SNVs (**Supplementary Table S4**). Notably, 8 SNVs that were consistently identified in both GCTA-COJO and FUMA analyses were defined as independent genetic risk factors for IBS.

### 3.5 Identification of genes associated with COVID-19 and IBS comorbidity

**Figure 2:**
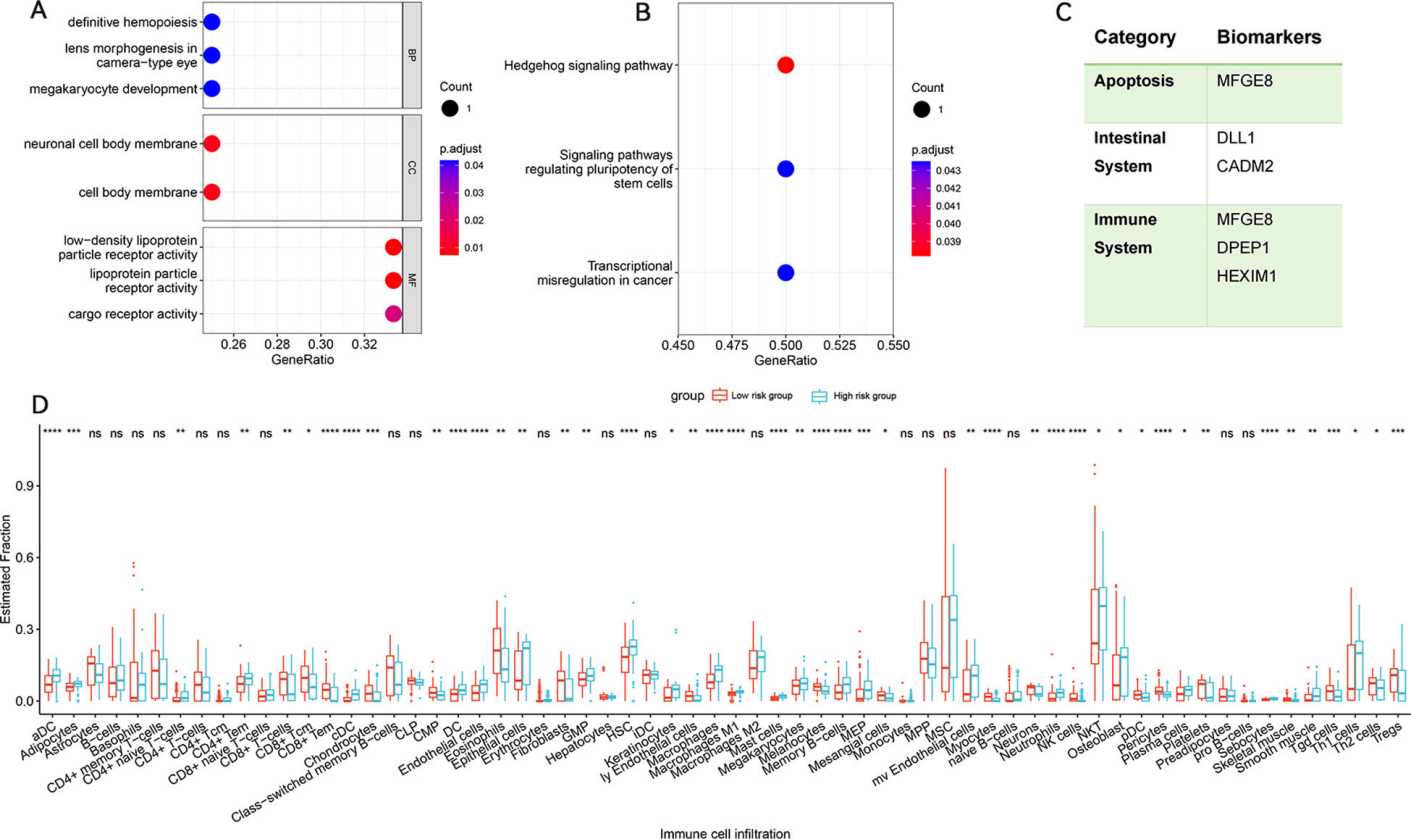
Enrichment analysis for identified risk genes. Significant Types of Pathways Based on GO **(A)** and KEGG Enrichment Analyses **(B)**. **(C)** summarizes the categories to which risk genes and proteins belong. Immune infiltration analysis of pleiotropic gene in IBS cohort. **(D)** Abundance of differences in immune cells between both groups in the IBS cohort. BP, Biological Process; CC, Cellular Component; MF, Molecular Function; KEGG, Kyoto encyclopedia of genes and genomes pathway. *p < 0.05, **p < 0.01, ***p < 0.001, ****p < 0.0001, ns, not significant.

MAGMA analysis identified 125 genes associated with IBS risk SNVs (**Supplementary Table S5**). Subsequent POPS screening highlighted 4 genes with POPS scores greater than 1 (**Supplementary Table S6**), marking them as potential IBS risk genes. SMR analysis, integrating GWAS summary data and eQTL information from various tissues and cell types, found that CADM2 was replicable in the SMR analysis (**Supplementary Table S7**). Genomic enrichment analysis indicated significant enrichment in pathways related to megakaryocyte development and the Hedgehog signalling pathway, suggesting their roles in IBS and COVID-19 (**Supplementary Table S8**) (**Figures 2A, B**).

### 3.6 Identification of proteins associated with COVID-19 and IBS comorbidity

Using the BLISS method, we identified 231 proteins associated with the comorbidity risk of IBS and COVID-19. Among these, four proteins (DLL1, DPEP1, HEXIM1, and MFGE8) were consistently found across three different databases (**Supplementary Table S9**), highlighting their potential as drug targets.

### 3.7 Identification and characterization of IBS subtypes

To identify IBS immune subtypes, we conducted single-sample gene set enrichment analysis (ssGSEA) to quantify the risk gene scores for each patient, categorizing them into high and low immune infiltration groups. Visualization of immune-related feature infiltration levels in bar plots revealed distinct patterns between these two clusters, showing low and high immune infiltration modes. The results indicated that Th1 cells, plasma cells, NK cells, neutrophils, mast cells, macrophages, and dendritic cells predominated in the high-risk group, whereas CD8+ T cells, eosinophils, and Tregs were more prominent in the low-risk group (**Figure 2D**).

## 4 Discussion

The complex genetic diversity observed in patients with COVID-19 and IBS required an approach beyond the traditional single-disease research paradigm, i.e. cross-trait GWAS analysis. Cross-trait analyses have demonstrated excellent validity in multiple co-morbidity studies (45–47), and this study aimed to elucidate the genetic underpinnings and complex interconnections between COVID-19 and IBS. The integration of GWAS summary data from two IBS datasets into a single META IBS dataset facilitated a detailed genetic association analysis of COVID-19. Significant genetic correlations were identified between IBS and both SARS-CoV-2 infection and hospitalization, enhancing the understanding of potential COVID-19 treatments. In a prospective COVID-19 cohort study, the OR for developing IBS in COVID-19 patients was found to be 12.92 (95% CI = 3.58-46.60, *p <* 0.001) (48), further indicating a potential interaction between the two conditions.

The integration of META-IBS with data from SARS-CoV-2 infected and hospitalized patients into a comprehensive multi-trait analysis was aimed at strengthening the statistical validation of the IBS dataset. This innovative approach led to the identification of three previously unrecognized significant genetic loci, markedly enriching our understanding of the genetic basis of IBS. Importantly, these SNVs had shown associations with COVID-19 in earlier studies, such as SNV rs10789340 (*p =* 4.23× 10^-17^), rs308523 (*p =* 1.32× 10^-4^), and rs61902812 (*p =* 1.06× 10^-9^), after adjusting the significance threshold to conventional statistical standards (*p <* 0.05). This result underscored the critical role of our research in advancing the identification of SNVs that were not deemed significant in previous studies, thereby deepening the understanding of the genetic foundation of IBS. A comprehensive gene association analysis incorporating MAGMA, PoPS, and SMR identified a correlation between CADM2 and IBS-COVID-19. CADM2, a member of the synaptic cell adhesion molecules, is involved in synaptic organization and plasticity (49). Although the precise function of CADM2 in IBS remains unclear, it is hypothesized that CADM2 might interact with the gastrointestinal tract through the enteric nervous system (ENS). The ENS consists of an extensive network of various intrinsic enteric neurons and glial cells, including motor neurons, intrinsic primary afferent neurons, and interneurons. Disrupted communication between enteric glial cells and neurons may contribute to ENS circuit dysfunction in IBS (50). Understanding the cell adhesion-mediated communication between glial cells and neurons in the ENS is vital for comprehending the role of ENS in both health and disease, warranting further research to clarify this mechanism.

The BLISS method was used to pinpoint four proteins associated with IBS risk, which were validated in the deCODE, UKBPP, and ARIC databases. Milk fat globule-EGF factor 8 protein (MFGE8) has been identified to exhibit characteristics similar to cluster proteins (51) and participate in pivotal biological processes such as apoptosis, immune regulation, and inflammation. MFGE8 also crucially maintains intestinal epithelial cell balance and promotes mucosal healing (52). Delta-like 1 (DLL1) has been characterized as a protein involved in the Notch signaling pathway, essential for the development, maintenance, and regeneration of the intestine (53). Abnormal expression of the Notch signaling pathway has been shown to inhibit the differentiation of intestinal epithelial cells, weakening the mucosal barrier and leading to IBS (54). Dipeptidase 1 (DPEP1), a zinc-dependent metalloprotease, has been recognized to process antibiotics, hydrolyze multiple peptides such as glutathione (GSH) and its conjugates, and be involved in the metabolism of leukotrienes (55). GSH is one of the most critical antioxidants in cellular biology (56), indispensable for upholding normal redox balance and antioxidant protection in the body (57). Reduced expression of DPEP1 can disrupt GSH homeostasis, thereby altering the redox state of the cellular microenvironment and protecting cells against pathological stress (58). HEXIM1, involved in transcription regulation, was found to regulate RNA polymerase II activity and the innate immune response mediated by DNA viruses by forming the HDP-RNP complex (59). **Figure 2C** summarizes the categories to which risk genes and proteins belong. While there is currently no definitive evidence linking these four proteins to IBS, our analysis suggests potential connections.

Evidence indicates low-grade inflammation and immune dysregulation involving various immune cells in the context of IBS (60). These immune cells contributed to IBS onset and progression through mechanisms such as cytokine production, alterations in the gut microbiome, immune cell infiltration, and effects on gut barrier integrity and nervous system function. Our immune infiltration analysis showed an increased proportion of mast cells, neutrophils, and M1 macrophages in the high-risk IBS group, while the proportion of regulatory T cells (Tregs) was reduced. Mast cells could be activated through IgE-dependent or IgE-independent pathways, releasing inflammatory mediators. An *in vivo* functional study of mast cell-deficient rats compared intestinal permeability between groups with and without mast cell inhibitors, revealing significantly increased intestinal permeability in the group without mast cell inhibitors (61). In addition, a case-control study (62) identified an augmentation in mucosal mast cell counts in colonic and duodenal biopsies from patients with IBS, specifically those who mainly presented with diarrhea symptoms. These findings underscored the role of mast cells in gut function and disease. Macrophages, highly plastic cells, could polarize into inflammatory (M1) and anti-inflammatory (M2) phenotypes (63). M1 macrophages released pro-inflammatory factors, including tumor necrosis factor-α (TNF-α), interleukin (IL)-6, IL-1β, IL-23, IL-18, and C-C motif ligand 2 (CCL2) (64). In IBS patients, the NF-κB/IκB-α pathway activation in mucosal macrophages fostered the expression of inflammatory factors like NLRP3, leading to increased secretion of IL-1β and TNF-α (65). Inflammation could cause visceral hypersensitivity and altered gut motility, worsening IBS symptoms. Moreover, the induction of inflammatory cascades and the accumulation of neutrophils could damage intestinal epithelial cells and form crypt abscesses, increasing mucosal immune cells and chemokines, and thereby raising intestinal permeability (66). Tregs, responsible for immune system regulation, when deficient or dysfunctional, were linked to various autoimmune and inflammatory diseases, including arthritis, IBS, and ulcerative colitis (67). This was consistent with our findings, thus supporting the reliability of the study.

## 5 Conclusion

Taken together, significant genetic associations between SARS-CoV-2 infection and COVID-19 hospitalization with IBS were identified, revealing new genetic risk factors and associated biomolecules. These findings enhanced the understanding of the genetic underpinnings of IBS and could inform the development of novel therapeutic strategies. Future research should aim to validate the biological relevance of the newly identified risk genes, such as CADM2, and proteins, including MFGE8, DLL1, DPEP1, and HEXIM1, to advance our understanding of IBS pathophysiology further. Nonetheless, the study encountered limitations such as the reliance on GWAS data predominantly from individuals of European descent, which may not adequately capture the genetic diversity of more varied populations. Additional research is required to confirm these results across a broader range of populations and to investigate the functional mechanisms behind these genetic associations. Additionally, due to the cross-sectional nature of the study, causal inference of COVID-19 to IBS is limited, and therefore, more prospective studies are needed for validation.

## Data Availability

The original contributions presented in the study are included in the article/**Supplementary Material**. Further inquiries can be directed to the corresponding authors.
